# Daily dataset of precipitation and temperature in the Department of Cauca, Colombia

**DOI:** 10.1016/j.dib.2023.109542

**Published:** 2023-09-03

**Authors:** Kevin Blanco, Sandra R. Villamizar, Alvaro Avila-Diaz, Catalina Marceló-Díaz, Erika Santamaría, María Camila Lesmes

**Affiliations:** a Universidad Industrial de Santander, Carrera 27 Calle 9, Bucaramanga, Postal Code 680002, Colombia; b Research Group ``Interactions Climate-Ecosystems (ICE)'', Earth System Science Program, Faculty of Natural Sciences, Universidad del Rosario, Carrera 24 #63C-69, Bogotá, Postal Code 111221, Colombia; c Universidad de Ciencias Aplicadas y Ambientales, Calle 222 #55-37, Bogotá, Postal Code 111166, Colombia; d Grupo de Entomología, Instituto Nacional de Salud, Avenida Calle 26 #51-20, Bogotá, Postal Code 111321, Colombia

**Keywords:** Spatial downscaling, ERA5-Land, CHIRPS, MSWX, Kriging

## Abstract

This study used the geostatistical Kriging methodology to reduce the spatial scale of a host of daily meteorological variables in the Department of Cauca (Colombia), namely, total precipitation and maximum, minimum, and average temperature. The objective was to supply a high-resolution database from 01/01/2015 to 31/12/2021 in order to support the climate component in a project led by the National Institute of Health (INS) named “Spatial Stratification of dengue based on the identification of risk factors: a pilot study in the Department of Cauca”. The scaling process was applied to available databases from satellite information and reanalysis sources, specifically, CHIRPS (Climate Hazards Group InfraRed Precipitation with Station Data), ERA5-Land (European Centre for Medium-Range Weather Forecasts), and MSWX (Multi-Source Weather). The 0.1° resolution offered by both the MSWX and ERA5-Land databases and the 0.05° resolution found in CHIRPS, was successfully reduced to a scale of 0.01° across all variables. Statistical metrics such as Root Mean Squared Error (RMSE), Mean Absolute Error (MAE), Person Correlation Coefficient (r), and Mean Bias Error (MBE) were used to select the database that best estimated each variable. As a result, it was determined that the scaled ERA5-Land database yielded the best performance for precipitation and minimum daily temperature. On the other hand, the scaled MSWX database showed the best behavior for the other two variables of maximum temperature and daily average temperature. Additionally, using the scaled meteorological databases improved the performance of the regression models implemented by the INS for constructing a dengue early warning system.

Specifications Table

**Table:** 

Subject	Environmental Science

Specific subject area	Application of Kriging methodology for the scale reduction of climatological data
Type of data	Downscaled datasets of precipitation and temperature for the department of Cauca (Colombia) at 0.01° resolution.
How the data were acquired	The scaled data was obtained by applying the geostatisical Kriging method to the National Oceanic and Atmospheric Aministration's (NOAA) CHIRPS database (precipitation), the ERA5-Land database (precipitation and temperature) from the Climate Data Store of the European Union's Earth Observation Programme, “Copernicus”, and finally, MSWX from the GloH2O web portal (precipitation and temperature).
Data format	Analyzed, nc (NetCDF) files
Description of data collection	The downscaled datasets were generated using the “KrigR” R-package, which uses the "automap" R package to carry out the Kriging downscaling process. KrigR was adapted in its three-step process to work for the three climate datasets by defining the target variable (precipitation or temperature), geographic area (-78° to -75.6° latitude and 0.9° to 3.4° longitude), time period (2015-2021) and temporal resolution (daily). A DEM in raw-data resolution and target resolution (0.01°) was used as covariate for the process.
Data source location	· Region: Cauca Deparment · Country: Colombia · Latitude and longitude for collected samples/data: -78° to -75.6° and 0.9° to 3.4°. · The raw climatic datasets are accessible on the following websites: • CHIRPS: https://coastwatch.pfeg.noaa.gov/erddap/griddap/chirps20GlobalDailyP05.html • ERA5-Land: https://cds.climate.copernicus.eu/cdsapp#!/dataset/reanalysis-era5-land?tab=overview • MSWX: https://www.gloh2o.org/mswx/ • IDEAM: http://dhime.ideam.gov.co/atencionciudadano/
Data accessibility	Repository name: HydroShare Direct URL to data: https://www.hydroshare.org/resource/3dbf521c03134a19a138d192bbe676d0/ DOI: 10.4211/hs.3dbf521c03134a19a138d192bbe676d0

## 1 Value of the Data

• The scaled database allowed the creation of high-resolution (0.01°) meteorological maps, which permit the description of the environmental conditions that promote the reproduction and survival of dengue vectors such as the *Aedes aegypti*, through ecological niche modeling.
• This information benefits institutions responsible for the surveillance and control of dengue, to avoid its propagation in the Department of Cauca.
• The daily downscaled data may be used in any study requiring high-resolution climatic information (precipitation and maximum, minimum, or average temperature).

## 2 Objective

A high-resolution database (0.01°) of daily climatic information (precipitation maximum temperature, minimum temperature, and average temperature) was created through the geostatistical Kriging method to support the National Institute of Health (INS) in the creation of an early warning system to monitor the risk of dengue in the municipalities of Patía, Piamonte, and Miranda, located in the department of Cauca, Colombia.

## 3 Data Description

**Fig. 1:**
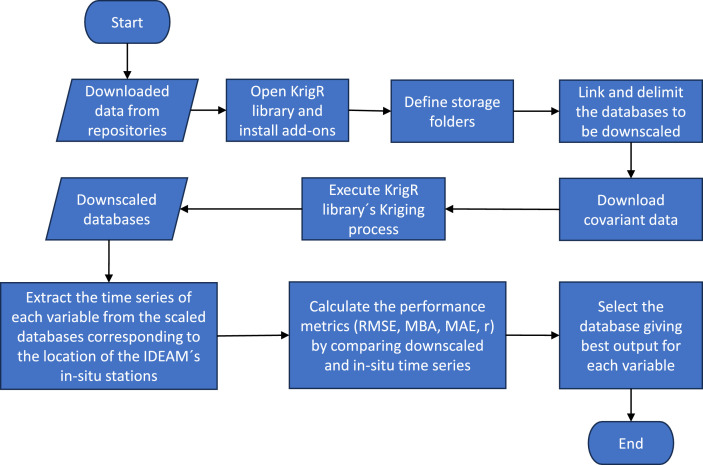
Scale reduction flowchart.

The Department of Cauca's scaled databases were generated by applying the geostatistical Kriging methodology to the CHIRPS, ERA5-Land, and MSWX databases. Fig. 1 shows a flow chart diagram that describes the method utilized in the scaling and evaluation processes and the selection of the high-resolution database that yielded the best performance in estimating each variable.

Table 1 shows the performance metrics used for the evaluation of the proficiency of the high-resolution databases in the estimation of the variables studied, in comparison to the in-situ data available from the network stations of the Colombian Institute of Hydrology, Meteorology and Environmental Studies (IDEAM).

**Table 1:** Estimated statistical metrics.

Name	Equation	Units	Perfect Score
Root Mean Square Error (RMSE)	$RMSE=\;\sqrt{\left(\frac{1}{N}\right)\sum_{i=1}^{N}{\left[P_{i}-Q_{i}\right]}^{2}}$	Precipitation (mm) Temperature (°C)	0.0
Mean Absolute Error (MAE)	$MAE=\frac{\sum_{i=1}^{N}\left|P_{i}-Q_{i}\right|}{N}$	Precipitation (mm) Temperature (°C)	0.0
Pearson Correlation Coefficent (r)	$r=\frac{\left(N\sum_{i=1}^{N}\left(P_{i}*Q_{i}\right)-\left(\sum_{i=1}^{N}P_{i}\right)\left(\sum_{i=1}^{N}Q_{i}\right)\right)}{\sqrt{\left[N\sum_{i=1}^{N}P_{i}^{2}-{\left(\sum_{i=1}^{N}P_{i}\right)}^{2}\right]\left[N\sum_{i=1}^{N}Q_{i}^{2}-{\left(\sum_{i=1}^{N}Q_{i}\right)}^{2}\right]}}$	–	1.0
Mean Bias Error (MBE)	$MBE=\frac{1}{N}\sum_{i=1}^{N}\left[P_{i}-Q_{i}\right]$	Precipitation (mm) Temperature (°C)	0.0

Where $Q_{i}$ represents the observed values for each meteorological variable (total precipitation, maximum, minimum, or daily average temperature) at one station; $P_{i}$ represents the values for each meteorological variable from the original or scaled databases (CHIRPS, ERA5-Land or MSWX); and N is the amount of data evaluated.

Fig. 3, Fig. 4, Fig. 5 show examples of scaled data for maximum, minimum, and daily average temperature from the ERA5-Land and MSWX databases compared to their original resolutions. Fig. 6 shows an example of the scaled data for daily precipitation from the ERA5-Land, MSWX, and CHIRPS databases. Lastly, Fig. 7, Fig. 8, Fig. 9, Fig. 10 present the results obtained for the estimated performance metrics.

**Fig. 2:**
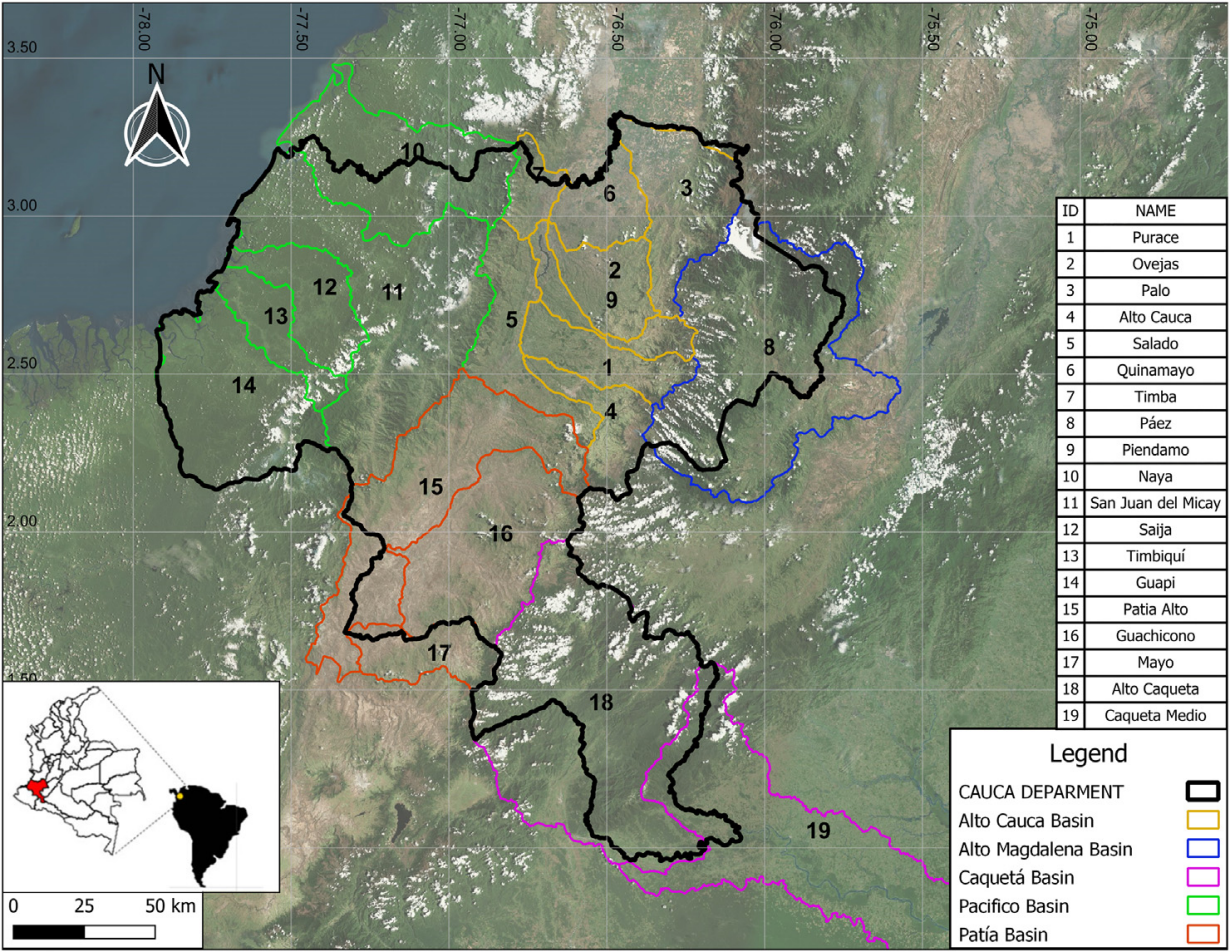
Study area location.

**Fig. 3:**
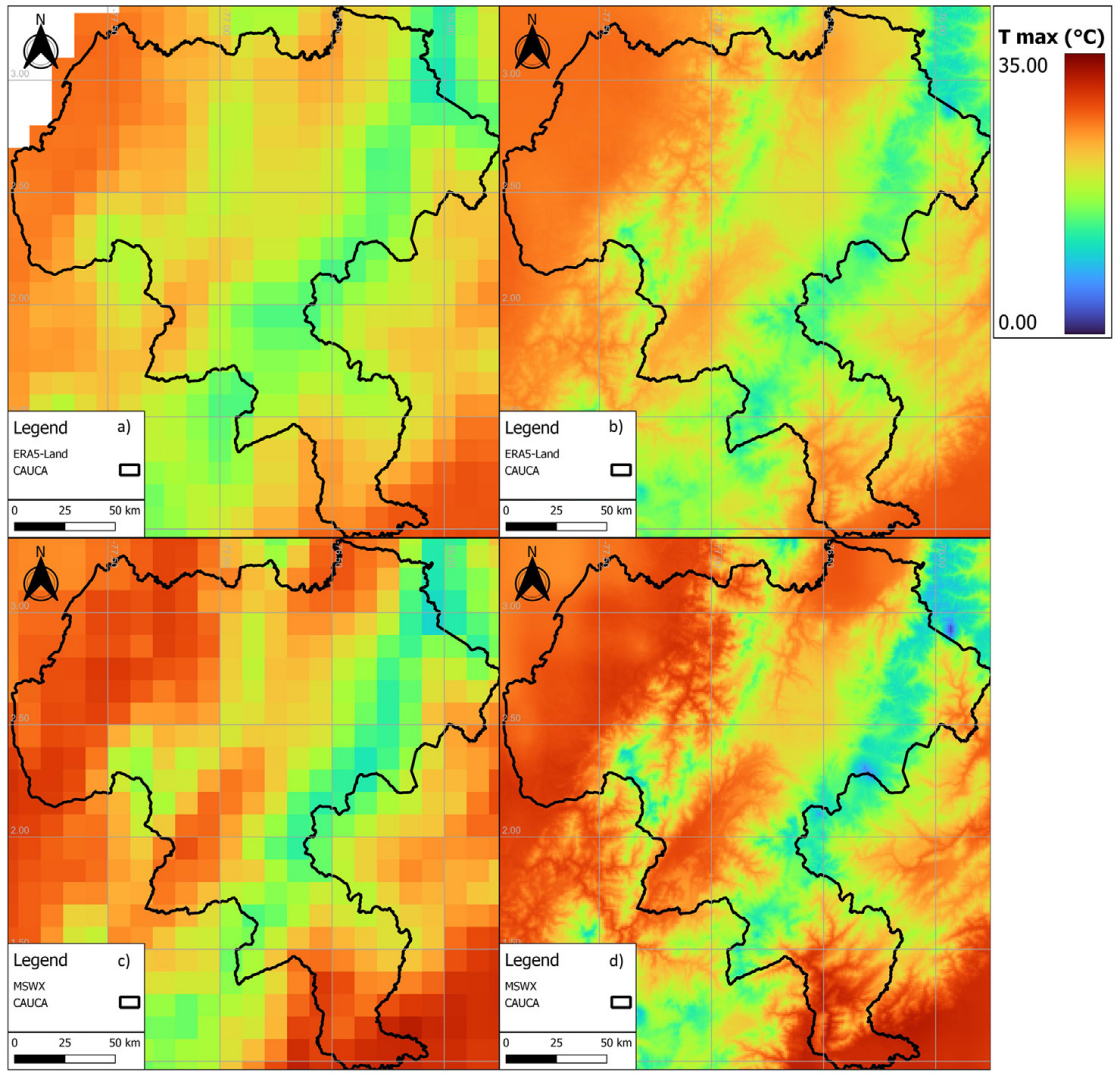
Daily maximum temperature for 01/17/2015. (a) ERA5-Land unscaled (0.1°); (b) ERA5-Land scaled (0.01°); (c) MSWX unscaled (0.1°); (d) MSWX scaled (0.01°).

**Fig. 4:**
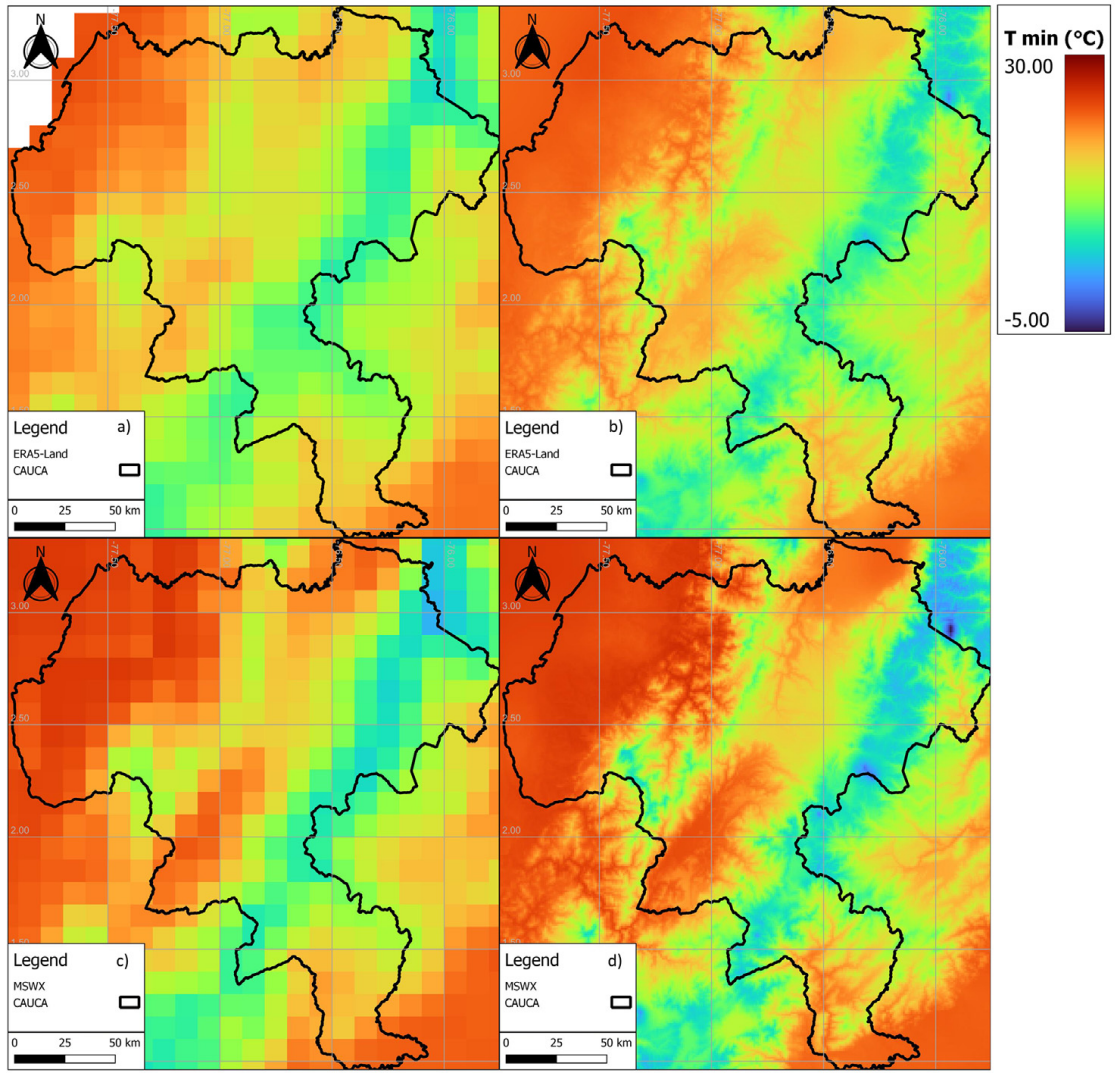
Daily minimum temperature for 01/17/2015. (a) ERA5-Land unscaled (0.1°); (b) ERA5-Land scaled (0.01°); (c) MSWX unscaled (0.1°); (d) MSWX scaled (0.01°).

**Fig. 5:**
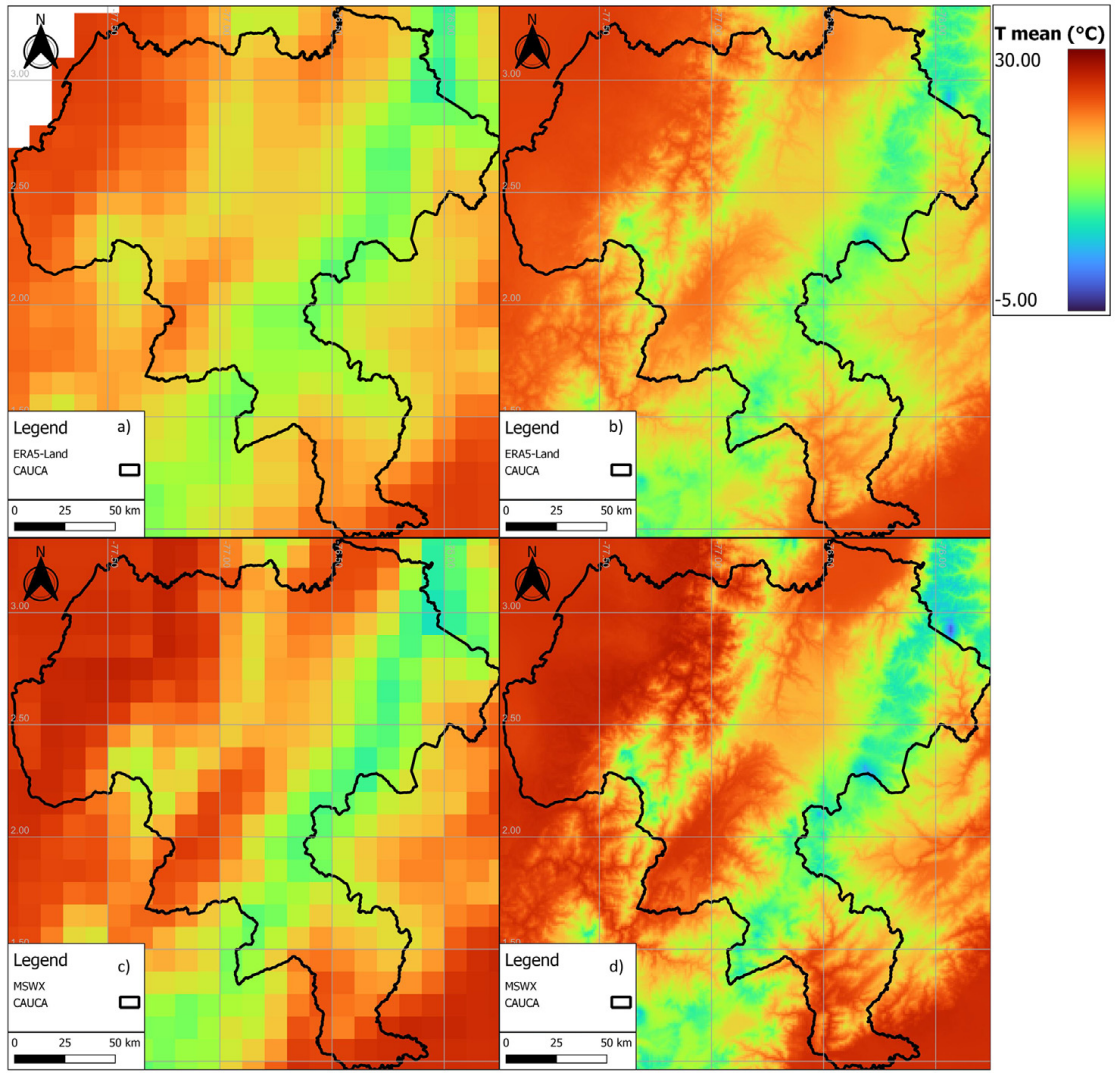
Daily average temperature for 01/17/2015. (a) ERA5-Land unscaled (0.1°); (b) ERA5-Land scaled (0.01°); (c) MSWX unscaled (0.1°); (d) MSWX scaled (0.01°).

**Fig. 6:**
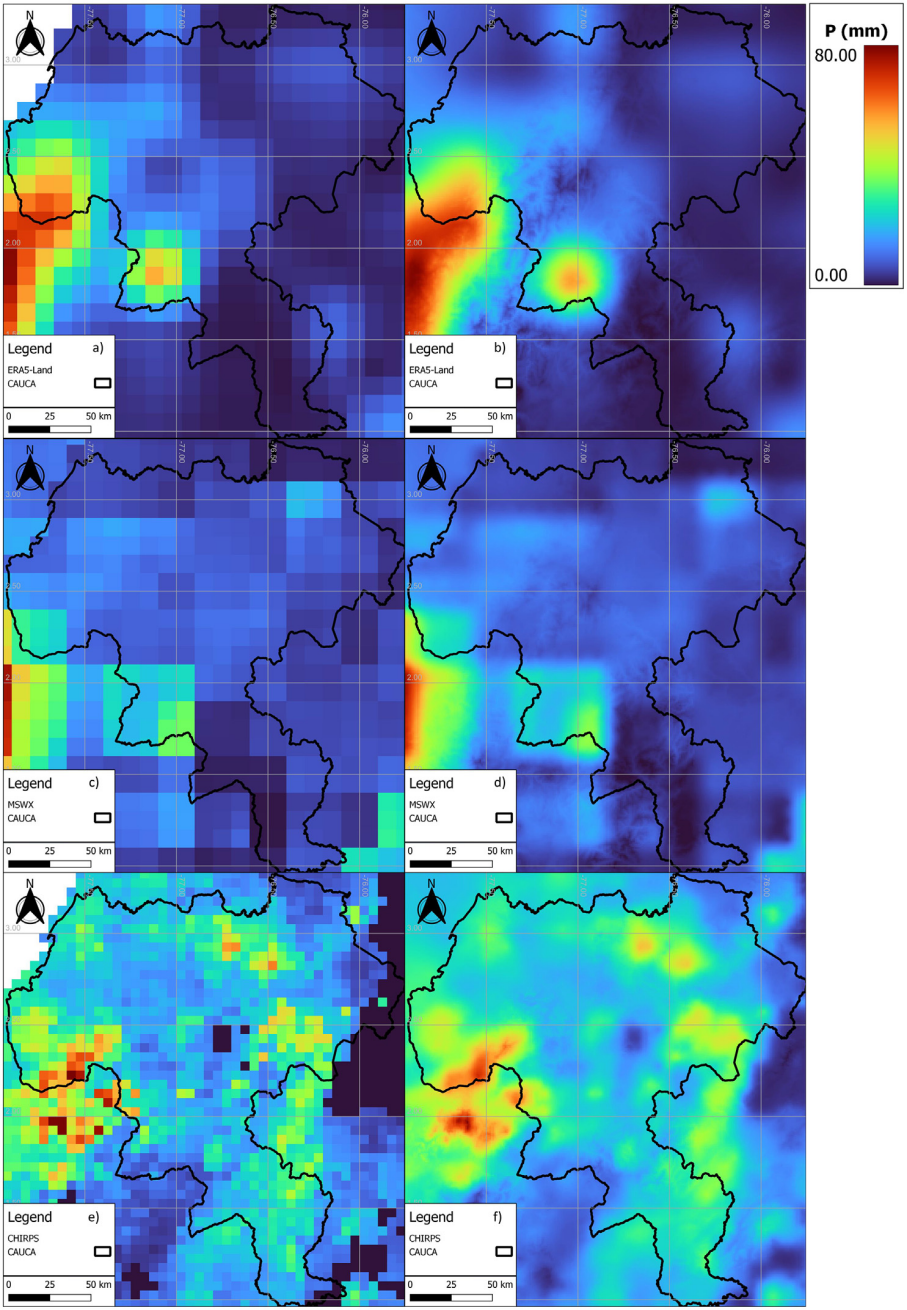
Daily total precipitation for 01/17/2015. (a) ERA5-Land (m) unscaled (0.1°); (b) ERA5-Land (m) scaled (0.01°); (c) MSWX (mm) unscaled (0.1°); (d) MSWX (mm) scaled (0.01°); (e) CHIRPS (mm) unscaled (0.05°); (f) CHIRPS (mm) scaled (0.01°).

**Fig. 7:**
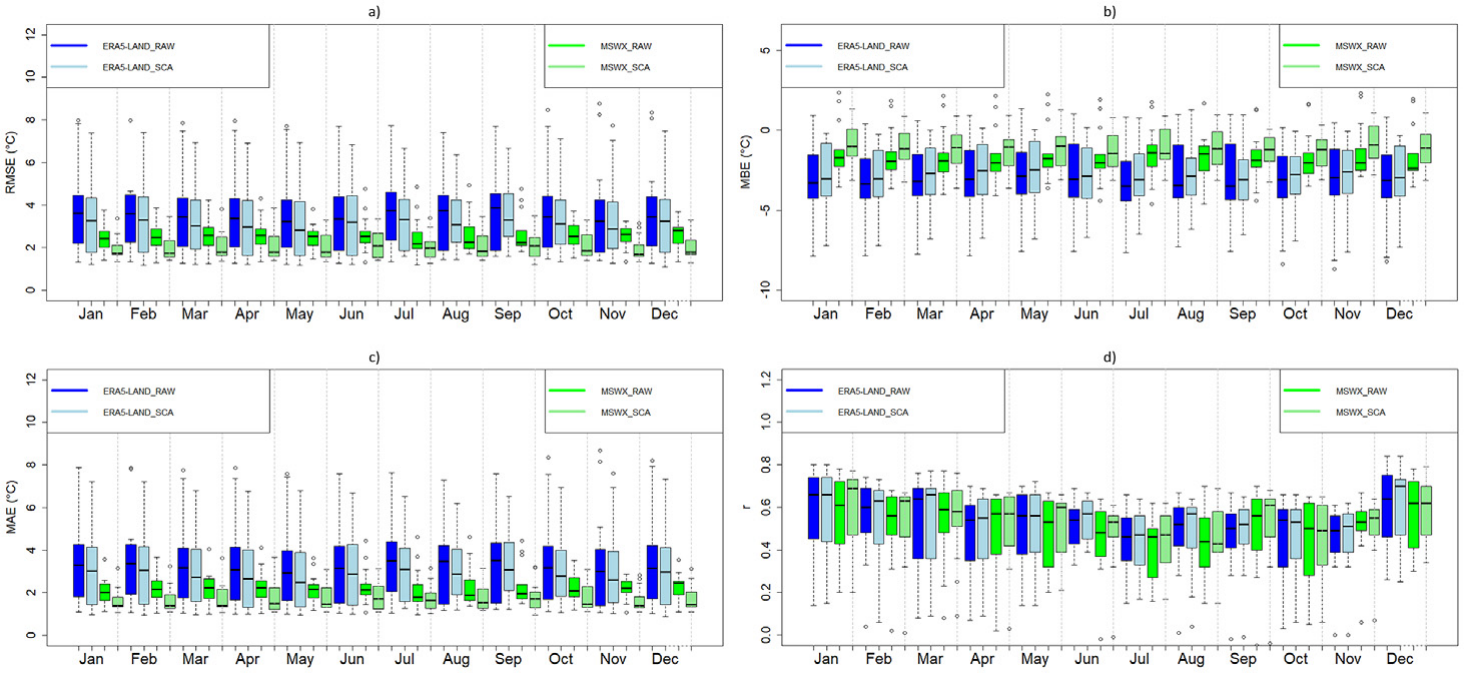
Daily maximum temperature metrics for the original and scaled databases. (a) RMSE; (b) MBE; (c) MAE (d) r.

**Fig. 8:**
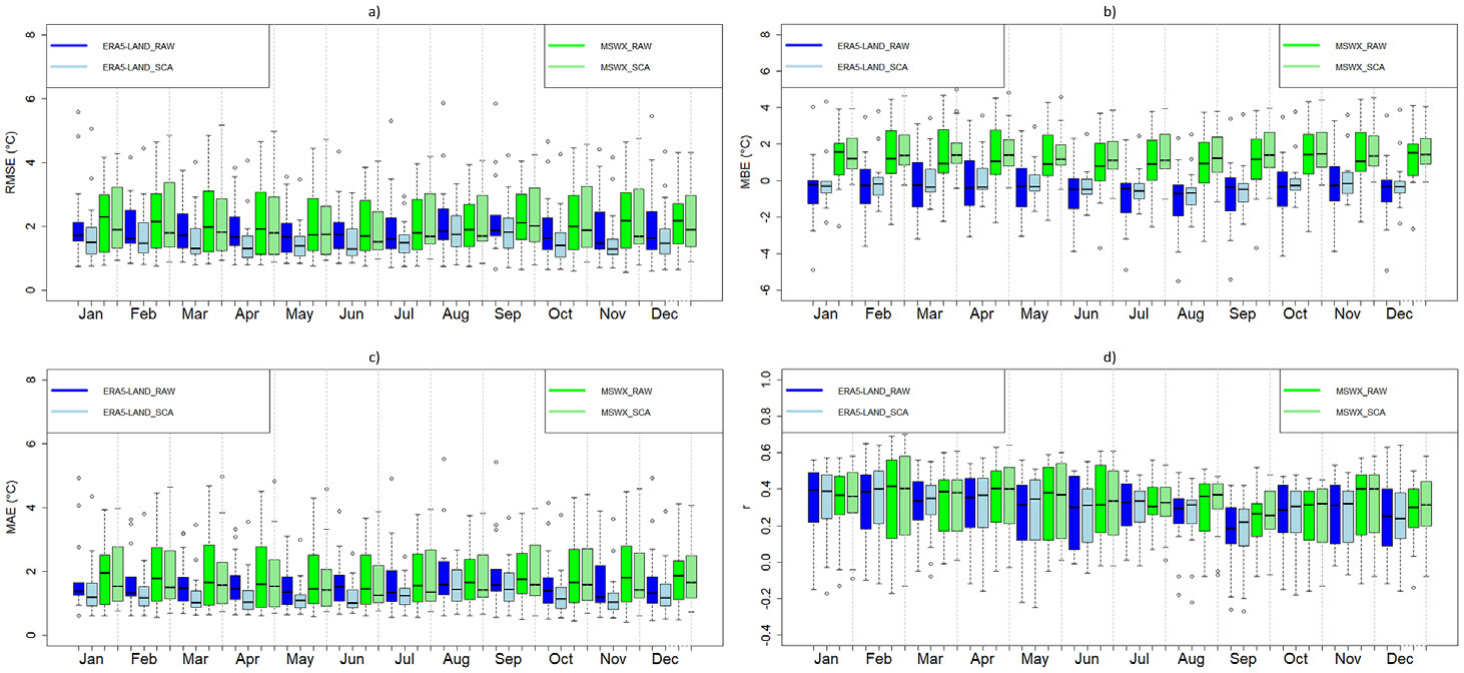
Daily minimum temperature metrics for the original and scaled databases. (a) RMSE; (b) MBE; (c) MAE (d) r.

**Fig. 9:**
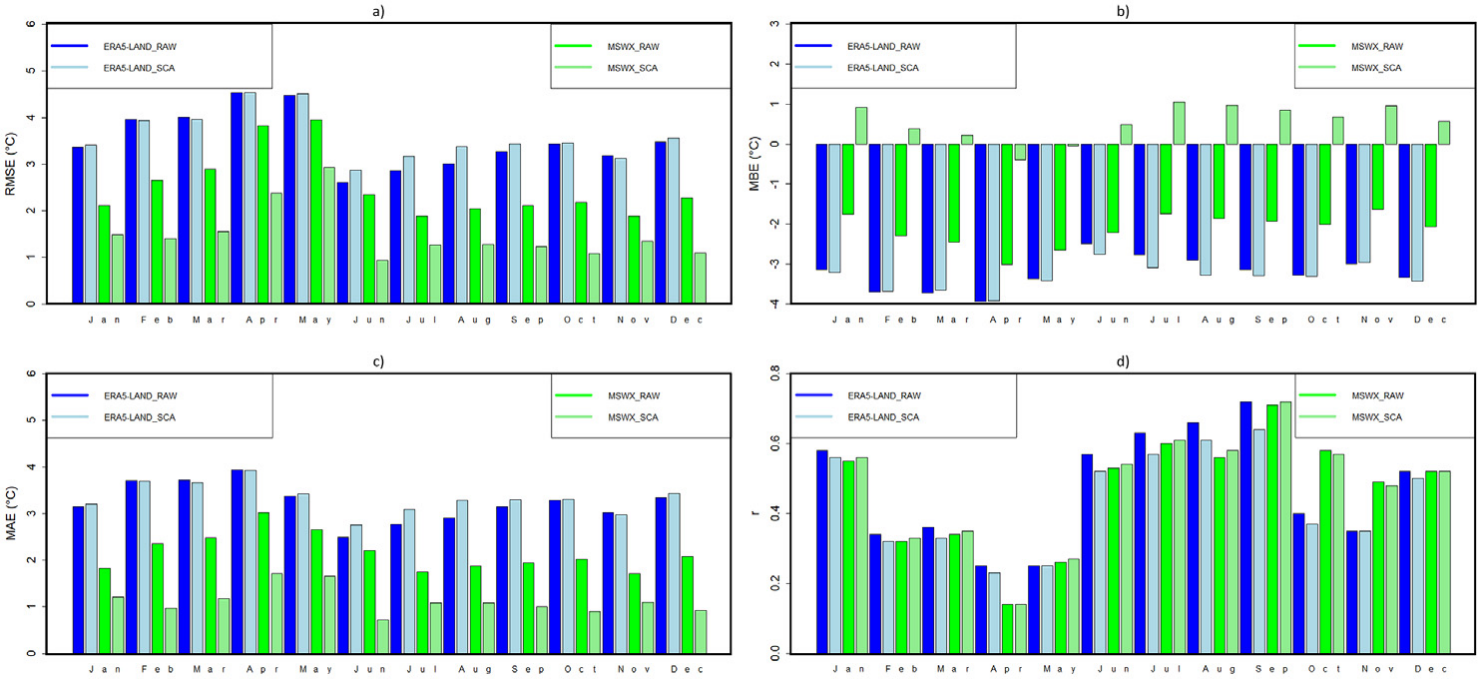
Daily average temperature metrics for the original and scaled databases. (a) RMSE; (b) MBE; (c) MAE (d) r.

## 4 Experimental Design, Materials and Methods

### 4.1 Description of the study area

In southwest Colombia, the Cauca department covers a surface area of 29,308 km² (Fig. 2). Five large watersheds can be found within its territory: High Cauca, Pacific, High Magdalena, Patía, and Caquetá. With an average altitude of 1693 m above sea level, the department presents a topographic profile that varies from 5750 m above sea level to sea level, which allows it to encompass all thermal floors. One can find a warm and humid climate in the Pacific coast to the west, while in the Patía watershed, the weather is warm and semiarid. In the central region, temperate humid and semi-humid climates predominate. The Popayán plateau has cold climates, and the *Nevado del Huila* sees perpetual snow. This diversity of thermal floors results from the altitudinal gradient that spans from the coastline to the most elevated mountainous zones [1].

### 4.2 Raw databases

The National Oceanic and Atmospheric Administration (NOAA) web portal was used to download the CHIRPS data [2]. This platform offers precipitation data which is derived from infrared observations of cold cloud durations; the pixel size of this dataset is 0.05°. The portal permits to define the time window, the temporal resolution, and the geographical area of interest, which, in our case, were the coordinates that encompass the department of Cauca (0.9° to 3.4° latitude and -78° to -75.6° longitude).

To access the data from the ERA5-Land database, a user account was created on the Climate Data Store website, which is part of the European Union's Earth Observation Program, (“Copernicus”) [3]. ERA5-Land employs a reanalysis process integrating data from climatological models with global observations. The pixel size of this dataset is 0.1°. We downloaded precipitation data and maximum, minimum, and average temperature data using the “download_ERA” function from the “KrigR” library in R, through the RStudio interface. This function allows to configure the time window, the temporal resolution, and the geographical area of interest within the script.

Lastly, before downloading the MSWX data (precipitation data, maximum, minimum, and average temperature data), it was necessary to complete the license request process on the GloH2O website [4], which is the institution responsible for developing MSWX. This dataset, with 0.1°-pixel size, consists of data generated from ERA5 that was corrected using high-resolution reference climatology. After the license request, an e-mail containing an access link to a Google Drive folder housed the data was received. Post-processing using CDO (Climate Data Operators) [5] allowed the extraction of the geographical area of interest (the raw files are for the entire globe) and to integration de individually downloaded daily files into the complete time window of the study.

### 4.3 Methodology

Kriging is a geostatistical method used for estimating spatial values in a specified area. It is based on the analysis of averages, covariance functions and variograms of the data available in said area. The method is widely used because it effectively allows interpolating data to finer resolutions by incorporating covariate information that helps determine the uncertainty associated with the downscaling [6], [7], [8]. Furthermore, this method ensures a notable reproducibility level, guaranteeing result consistency and the ability to replicate findings. There are three main forms of linear Kriging: simple Kriging, which utilizes linear estimators with a known mean and a covariance function; ordinary Kriging, which is used when there is no known average and utilizes only covariance functions; and universal Kriging, which employs variograms to construct the estimator [9]. This project used the universal Kriging to carry out the estimations.

$$\sum_{\beta }\lambda_{\beta }\gamma_{\alpha \beta }+\sum_{l}\mu_{l}f_{\alpha }^{l}=\gamma_{\alpha 0}\;\alpha =1,\;\ldots .,\;N$$

$$\sum_{\alpha }\lambda_{\alpha }f_{\alpha }^{l}=f_{0}^{l}\;l=0,\;\ldots ,\;L$$

Where λβ is the weight of the dataset at point β, ϒαβ is the variogram between the datasets at points α and β, $f_{\alpha }^{l}$ are the known base functions of the dataset at point α, μl are L + 1 unknown variables used utilizing Lagrange multipliers, ϒα0 is the variogram between the dataset at point α and the dataset at point 0, λα is the weight of dataset α, and $f_{0}^{l}$ are known base functions of the dataset at point 0 [9].

A variogram can be mathematically defined as “Half of the mathematical expectancy of the square of the difference of two values of the study variable Z, corresponding to positions separated by a distance of h” [10].

$$\gamma \left(h\right)=\frac{1}{2}E{\left[Z\left(X\right)-Z\left(X+h\right)\right]}^{2}$$

Where E[Z(x)] is the mathematical expectancy operator, X is a vector of coordinates, and h is the distance that separates the two points where variable Z is measured [10].

The implementation of the geostatistical Kriging method in the scale reduction of the study's climatological variables of interest used the krigR function (multi-core Kriging) of the KrigR library found within R. This function requires training data which are to be scaled, along with covariable data in two resolutions: the resolution of the training data and the target resolution to be reached by the scaling process. From this information, the scaling procedure is carried out in two steps; first, the tool adjusts variograms to the training dataset and establishes functions of covariance with the covariable data in the resolution of the training data, generating functions that make it possible to describe the spatial correlation between the training data and the covariables [6]; second, the prediction of the values of the variable in the new location is carried out, parting from the covariable data in the target resolution [11]. In the process of scaling the databases, the digital elevation model (DEM) (0.01° resolution) from the United States Geological Service (USGS) was utilized as covariant information [12].

### 4.4 Validation

The scaled databases were validated by using a comparison between the downscaled data and the in-situ measurements at the local IDEAM station network [13].

Obtaining the in-situ measurements for the comparison involved consulting and downloading the information available at the local stations in the department of Cauca for each of the meteorological variables of interest in the period between 01/01/2015 and 31 /12/2021 (which corresponds to the period of the study carried out by the INS). The data initially downloaded in comma-separated values (.csv) format contained daily values for total precipitation and maximum, minimum, and average temperatures. Subsequently, the data sets were processed through Microsoft Excel to identify and eliminate erroneous data, specifically, values that had typographical errors, and to verify the temporal continuity of the time series, which allowed the identification of a total of 12 stations for the analysis. validation of precipitation data, 13 stations for the maximum temperature variable, 14 stations for minimum temperature and only one station for the case for the average temperature. It should be noted that, since the IDEAM data was used to assess performance in the original and scaled databases, the missing data-filling procedure was not performed. This was done to avoid introducing errors associated with this procedure to the estimated metrics (Table 1) for the evaluation of this study.

**Fig. 10:**
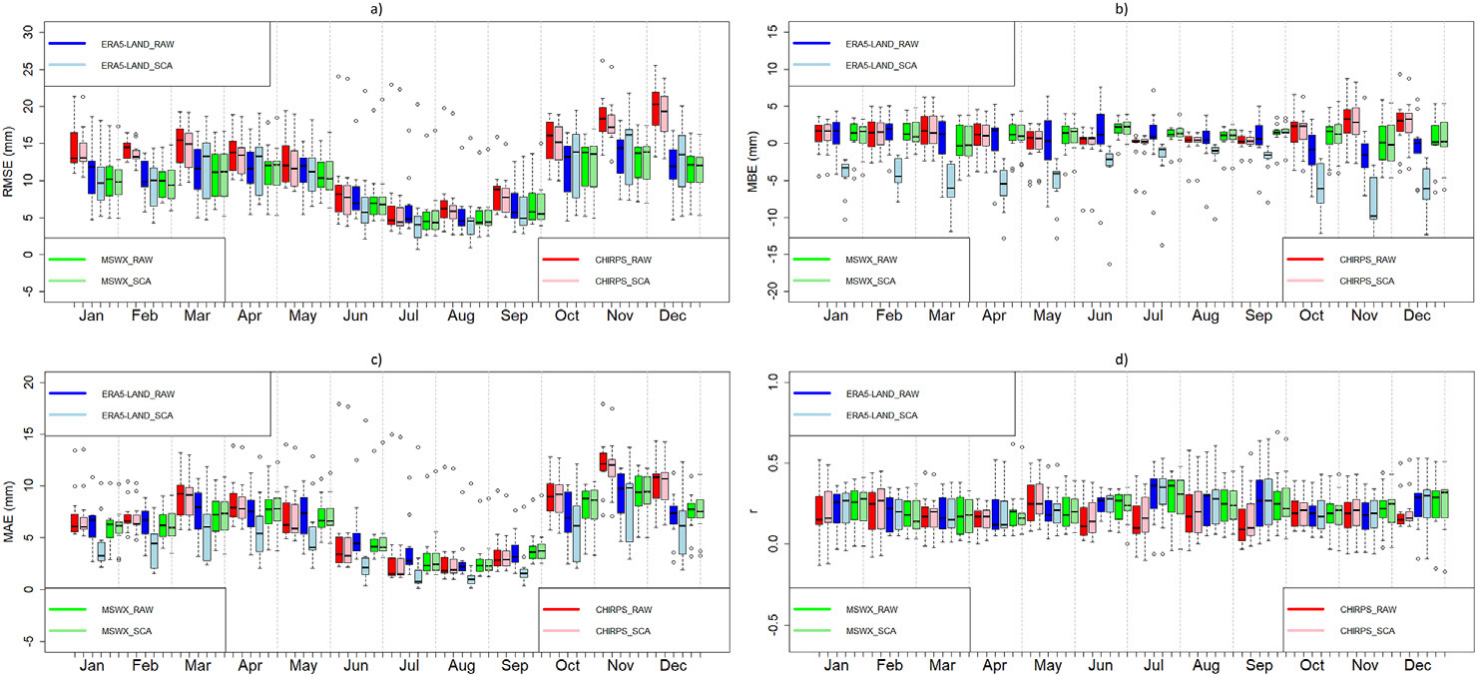
Daily total precipitation metrics for the original and scaled databases. (a) RMSE; (b) MBE; (c) MAE (d) r.

From the computed performance metrics (RMSE, MAE, r & MBE), it was determined that the scaled ERA5-Land databases yielded the best performance for the variables of precipitation and daily minimum temperature. On the other hand, the scaled MSWX databases obtained the best results for daily maximum temperature and daily average temperature (Fig. 10).

## Ethics Statements

This paper adheres to the ethical requirements set forth by the journal Data in Brief for the publication of summary data.

## CRediT authorship contribution statement

**Kevin Blanco:** Conceptualization, Methodology, Software, Data curation, Writing – original draft, Visualization. **Sandra R. Villamizar:** Conceptualization, Methodology, Software, Data curation, Visualization, Writing – review & editing. **Alvaro Avila-Diaz:** Conceptualization, Methodology, Software, Data curation, Visualization, Writing – review & editing. **Catalina Marceló-Díaz:** Supervision, Writing – review & editing. **Erika Santamaría:** Supervision, Writing – review & editing. **María Camila Lesmes:** Supervision, Writing – review & editing.

## Declaration of Competing Interest

The authors declare that they have no known competing financial interests or personal relationships that could have appeared to influence the work reported in this paper.

## Data Availability

Daily Database of Precipitation and Temperature in the Department of Cauca, Colombia (Original data) (HYDROSHARE).
